# Discussion of Papers on Filling Materials

**Published:** 1905-12-15

**Authors:** 


### DISCUSSION OF PAPERS ON FILLING MATERIALS.

Dr. Parmenter: One point which was not brought out very clearly and which I think ought to be made more of, is the polishing of amalgam fillings. I think we ought to put just as nice a finish on every amalgam filling as we would on a gold filling, for several reasons. A well polished filling causes less trouble with food collecting on its surface, and whenever a filling begins to give way around the margin it is much more easily detected if the amalgam fillings are polished smooth than if they are left as they are crystalized when not polished. If we have an examination of our fillings once or twice a year, it is much easier for us to determine what fillings are giving out. If they are left rough we can never know what fillings are commencing to leak, and they may leak quite a bit or almost come out before they are discovered. The first amalgam filling that I believe I ever saw polished I saw as the work of a dentist in Butte, Montana. That was five or six years ago, a bicuspid in which the contour had been nicely restored and the tooth was nicely finished and I said, that dentist takes pride in his work. Since then I have polished all my amalgam work, and find the results much more than repay the extra time and expense. I find that my patients are willing to pay an extra fee for that kind of work, and it is much more satisfactory.

Dr. N. S. Hoff: I am very much pleased with these papers. They have resulted in exactly what I had planned, and I should congratulate myself on the selection of the men to present these subjects, because they have presented exactly what I think we need, and the subjects have been presented from their respective standpoints as strongly as perhaps you will have an opportunity to hear them. I would like to carry that prophylactic idea along this same line of work. I do not know what is to be the future of the porcelain inlay or amalgam. I hope and expect that we shall have all of these materials at our command and will make use of them for a long time, and I suspect that if this prophylaxis treatment comes into public favor, as it seems now destined to do, that we shall make better fillings with both amalgam and gold than we are now making. I also anticipate that the next few years we will be putting more porcelain fillings in the posterior teeth than any other kind. This may seem strange to you, and it may seem as if that is a mistaken statement to make. We are testing out this porcelain inlay filling at the present time. I think if it is possible to insert porcelain fillings in the anterior teeth and they will stand the pressure, we will be led to make these fillings, and if we can do this, there is no filling, in my judgment, that is better adapted to assist and help on this subject of prophylaxis than porcelain. It is the filling which has an ideal surface, a surface which will not accumulate the extraneous materials from the mouth or produce infection of the gum. It is the one filling that it seems to me has a future in that direction if it is practicable to adapt it to the bicuspid, molar and bicuspid teeth; it will then be adopted by all the better practitioners simply from this standpoint of prophylaxis. I anticipate that this matter of prophylaxis is going to be, in the future, one of the most important parts of our practice.

It is not going to be everything; it will not do away with the present filling materials, but we will shift our methods of depending upon gold, for instance, which has always been the standard of the skillful operator, to porcelain. The scientific researches that are being done upon amalgam as a filling material, and the great effort which is being made now to develop better filling materials, will result in an ideal filling material. If this prophalactic ideal prevails, and men are induced to make more careful operations with amalgam and more careful observation of these teeth and place them in proper form that they may be cleaned easily and kept clean by the mouth toilet by the patient, amalgam has come to stay also, and we shall have three excellent materials with which to do filling work. I think, with Dr. Zederbaum, that amalgam has its place, and the amalgam of the future is going to be vastly better than the amalgam of the past. I do not believe it is possible for any one to insert gold in the posterior teeth, particularly in mesial surface of the lower molar teeth, and to polish these fillings so that it is possible to keep them free from extraneous deposits, it is however practicable to do that with porcelain. It is easier, it seems to me, to secure smooth surfaces there with amalgam. If we ever secure an alloy that is hard and dense and will retain its position, and not flow from the physical changes which take place in it, causing it to bulge out or pull away from the margin, and which will make a close joint which can be highly polished, I do not know but what it may supersede gold, and thus we may get beyond the gold stage. I am a gold operator myself and have tried to make just as good gold fillings as I knew how, but I believe we are going to do less gold work in the future than we have been doing. The future amalgam filling is going to be a much more careful operation than it has been in the past; we are not only going to take more care, but we shall use better material and be more particular about the kind of alloy we use, and we shall surely get better results. If these things come to pass, they will not put us out of business, we may not have more, but we shall have better amalgam fillings.

Dr. A. M. Long: I would like to take an exception to one statement in Dr. Sweetnam's paper, in regard to the age limit of 18 for gold. I have seen patients sixteen years old, or even fifteen, for whom I believe you can make a good gold filling, and I have seen people twenty-two and twenty-three in whom I would not risk a gold filling. I think the individual tooth has got to be considered more than the age. When I find a patient that the teeth are soft and I have commenced operating, I frequently put in cement to carry that tooth along until it goes through a hardening process, which it will do under a cement filling. If we take *more care* to examine the condition of our patient's teeth when they are presented to us, we will do better work and make better fillings. No mouth should be dismissed without being properly cleansed. Not only the amalgam fillings that we make to-day but the amalgam fillings that have been made eight or ten years ago, a great many of them will expand and bulge out and require polishing just as much as if they had been done recently. We should go all over the mouth and take care of it. Those old fillings often call for our immediate attention just as much as the new ones. I do not know how to estimate the porcelain fillings. I know of people that have had them in a good many years. I have some in twelve years and I am proud of them to-day. I believe that every dentist in this room would like if he could to dispense with gold. It is a precious metal and we have enjoyed it for years, if we could use less gold there would be a good deal less gold go to the grave yards. If we can discover something that we can use easily and make it do our patients more service than gold or porcelain, we should do so and save our patients the expense.

Dr. W. H. Jackson: Any filling's life of usefulness is no longer than its weakest point. Whatever filling you use depends very much upon the condition of the teeth and mouth in which it is placed. As far as my own experience goes, a perfectly placed gold filling is the most lasting and the best preservative of the teeth of any material I ever used. On the other hand, were it possible to take that gold and put it in a tooth in which the mallet or condenser would in the least particle displace any portion of the enamel in which it is placed, we might make an opening for the secretion to soak through the tooth and ultimately destroy the filling, and the tooth would be worth nothing. The weakness of a gold filling is due many times to improper manipulation, because we know that the secretions do not disintegrate the gold. The weakest part of the porcelain filling is the cement that holds it in the cavity. Find a cement that the secretions of the mouth will not act upon, and the porcelain inlay will be the ideal filling. There are persons in whose mouths cements will last eight, nine or ten years, but they are very scarce; on the other hand, you may put cemet fillings into the mouth without the porcelain covering, in a year's time all of them will show much solution and may need replacing entirely. We seldom see a mouth in which this is not true. If the saliva will act upon a cement filling, why should it not dissolve the cement filling which it is between tooth and porcelain. The inlay will last just so long and the cement will hold, and no longer. I have seen the work of some of the most expert operators, and some of the finest work that I ever saw in my life has failed in three years. Some inlays may last ten years, while as we may expect, others last not more than one year, because the cement dissolves out. I have seen amalgam fillings that have lasted forty years, and they are in yet, and I have seen others that did not last three years. It is the intelligent judgment of the man that is doing the work that counts, the man who uses proper discretion in selecting for the place that which is proper. The same amalgam put in one person's mouth may last forty years, while in another person's mouth it may not last three years. The secretions of one mouth will act upon the filling whereas in another mouth they will not. We should be able to discern what will last in one mouth and what will not in another. We should by knowledge and experience be able to recognize conditions which are detrimental to every kind of filling, and discriminate in our selection,

Dr. M. L. Ward: I would like to call your attention to one thing in regard to shrinkage of amalgam. Dr. Jackson has just called your attention to the fact that you could get good results in one mouth and not in another. There are reasons for this which are not in the manipulation or material. Alloys should have plenty of copper in them, the sulphite of copper is strongly antiseptic. Until ten years ago the cause of shrinkage was not determined, and it was not eliminated until that time. One of the first and important things in using amalgams is the selection of a proper alloy. It is easy to use an alloy with ten or eleven percent of copper to save all kinds of teeth. There are other things in the use of amalgam which ought to be recognized, amalgam fillings should be frequently polished. You can not expect any of the alloy fillings to retain a high finish. There is tin in all the alloys, which will not keep a high polish. You can not expect one to hold a polish longer than one year, even though it may have a high percentage of silver. It will not be as permanent as a gold filling because it contains more or less mercury.

Dr. J. Q. Byram: It seems to me to a large measure that this matter of filling materials resolves itself into the personal equation—what one man may do with a certain filling material may not be accomplished by another. Dr. Raymond spoke of one of the disadvantages that is claimed for porcelain inlays is the difficulty of manipulation. I am frank to say that it is easier for me to construct a porcelain inlay than it is to insert a gold filling, while with many it is much easier to insert a gold filling, and, as Dr. Jackson said, no filling is stronger than its weakest point, I wish to say that that is the greatest advantage of the porcelain inlay.

I dare say if we would examine all the gold fillings that have been inserted in the mouths here present, and all the amalgam fillings, that we should find at least seventy-five percent of them probably that are faulty. We consider a filling perfect when it is inserted and looks well. When we examine this filling carefully, and especially the amalgam filling we find there are places where the filling leaks, and we know too well that we should have our patient return for repair, but we leave them year after year with fillings that in our hearts we wish were out, but we placed them and we haven't the nerve to tell the patient that the filling is faulty and should come out. With the porcelain filling as soon as that filling is faulty you do not need to tell the patient the filling is faulty; he comes with it in his hand and to me that is one of the greatest advantages of porcelain as a filling material, the fact that the patient knows immediately when the filling is faulty. We are then compelled to reset the inlay in order to preserve the tooth. I am willing to say that I believe that the inlay for the purpose of filling teeth is the correct principle, and I believe that the time will come when practically all teeth will be filled by that method, whether it be gold or porcelain. I believe that we can retain inlay fillings better and without great sacrifice of tooth structure. In the inlay we have a filling that is less of an irritant to the pulp than when we rely on retention by carving grooves and retaining pits for the filling by the old methods.

I am sorry that Dr. Sweetnam took the stand that he did about non-cohesive gold, because I am a believer in non-cohesive gold. When I see the work that was done by such men as Taft, Keeley, Hunt and others years ago, it fills me with chagrin that I can not do as well. Now he said that that was probably not due to the filling material but to the individual. I agree with him to a large measure but I believe the same gentlemen could have taken any, other material, were it possible, and use it to as good an advantage and obtain the same results. And I also wish to say that I am an ardent advocate of hand pressure for gold filling. If I had my way about it, the manufacturers of automatic pluggers would go out of business. I think that more gold fillings are spoiled because of the fact that the margins are battered with the pluggers used in automatic pluggers than is done in any other way, and if we should take the pains to insert gold fillings by hand pressure as we do other fillings, we should get the best gold fillings. I also believe that we shall always have three filling materials, in amalgam, gold and porcelain. While, as I said before, the inlay principle will be the future practice, there will be cases where it is not advisable, for instance, in extremely small fillings I should consider it not advisable to use it. There are cases where amalgam should be used because of the inaccessability of the cavity. I gave you my opinion as to porcelain this morning for bicuspids and molars. I quite agree with Dr. Hoff that it is the best filling to aid prophalaxis that can be used, and were it possible to overcome its weak point, this edge turning, then it would be the ideal filling for bicuspids and molars; and I will simply reiterate what I said this morning, and say that the nearest way to overcome that is to sacrifice enough tooth structure to carry the margins to a point where there is practically no strain applied.

Dr. T. J. Collins: We do not want to be led by the enthusiast; we want to be led by men of judicial minds. I cannot see why any man should stake his all on the gold filling, or on the porcelain, or on the amalgam filling. Regarding the porcelain inlay, I believe that in the hands of skillful men it is good. A porcelain filling in many cases is useless and will not stand. There is one point that I want to bring before this society, it is with reference to the cost feature. It has been stated here to-night that the cost of the filling very often determines the use of amalgam. Should not the cost of the filling dominate, to a large extent, the kind of filling that is used? If you make a small inlay you can put it in at a less price than a large inlay; but a small gold filling you cannot. Are not a great many amalgam fillings made for the mere reason that they can be made at a less cost? I belief that the fee we receive to a large extent compels the use of amalgam for filling many teeth that would be filled otherwise if the fee were different.

Dr. C. H. Oakman: In regard to automatic pluggers. At every examination held by the State Board, each candidate for examination has from one to three automatic "pluggers," and in their operative work the success of their examination is largely due to those automatic pluggers. When it is not possible on a soft tooth to get a good margin and you use an automatic plugger, the first piece of gold that goes in is likely to see a piece of the tooth chipped off. I saw the senior class in Ann Arbor operating. They were cautious and used the hand mallet and hand pressure, and I said: "Boys, don't you use automatics?" They said: "They do not allow us to have them." I daresay that we have men here to-day that can talk in favor of the automatic plugger, and I hope to hear from them. Expert operators do the best work by the use of an automatic plugger, but uless they are experts in its use, it is not a good thing toe unse. I do not believe that we can all use hand pressur and make the same degree of success; one man will have strength in his wrist and can put gold down that the mallet will not change in the least. Where one man can do that, there may be ten who cannot. I insert a good many amalgam fillings, and lots of them I wish I had not inserted, I do not think there was ever an amalgam filling put into any tooth that did not sooner or later shrink or change form. The question we all ask is, what are the elements required in the perfect alloy, and where shall we get it? If we must use amalgam we should know what it is made of and who makes it. A good formula well manufactured, can be used to good advantage.

Dr. J. A. Watling: I have two amalgam fillings in my mouth that have been there 49 years next September. The way that amalgam was made I will tell you. A silver quarter was filed into filings and then mixed with the mercury, tin foil was added, and that alloy made those two fillings. One of them is in a cavity of the second molar, and another on the distal side of the first bicuspid. Has there been any improvement in amalgam since that day? Have we any amalgams to-day that will stand the test of 49 years? If there are any, you have not proved it by the use of them, and I doubt very much whether any new researches are going to improve amalgam over what it was 25 years ago. Amalgam is simply a combination of the metals, tin, silver, copper and mercury, mixed together. If the fillings are put in properly and in proper places they will last for many years. These fillings of mine were put in 49 years ago, long before the days of rubber dam, absorbent cotton or anything of that kind. The cavities were simply cleaned out by hand and wiped out by commercial cotton, and the fillings are good yet and will last to the end of my days, I have no doubt. I do not think there will be anything gained by the investigation of amalgam composition. We read in the journals about amalgam being absolutely a perfect filling material. One of the most advertised amalgams of recent years which is sold at the high price of $3.00 an ounce, never cost more than 50 cents an ounce to manufacture. The advertisement states that it is an absolutely perfect filling, and yet I do not believe there will ever be many fillings made of it that will last 49 years.

Dr. G. W. Shattuck: Standing here this afternoon listening to these arguments, the question arose in my mind whether we are going ahead or going back, or whether we are standing still. Over forty years ago there was held a convention out on Woodward Av., it was the old American Dental Convention, and if I could get hold of a copy of the proceedings and read you some of the discussions I think you would find them to be about the same as has taken place here this afternoon. Always this same question. I do not see that we have made any progress. What great things we are going to do remains for the future to find out. We still have our gold, cement, amalgam. We saved teeth forty years ago with these materials; we have examples of that to-day. With our hand drills we used to drill out the cavity. I first saw rubber used in 1864 at this convention. Last night I was surprised at what was brought out under the discussion of prophylaxis. We used to have a little bit of a stick of pine or orangewood, and took the pumice stone and pounded it out on a bench or anvil and made our powdered pumice stone. We had to make our scalers of bar steel which we put in the flame and tempered. With these we scaled and polished the teeth.

Dr. C. H. Worboys: I should prefer to speak of the advantages of inlays, both of porcelain and gold, but I will refer to the discussion of other fillings. One of the reasons why some our amalgam fillings fail so often is that we do not cut away weak walls enough. Too many of us are using the engine in place of a good heavy chisel. The same with gold fillings. You cannot make a gold filling stay where there is any stress applied to it unless you can thoroughly condense the gold into the retaining form of the cavity, and that gold must be cohesive gold on soft gold, non-cohesive gold will not hold. A gold filling properly made must be like a cork, just as perfect as it is possible to make it. My friend Oakman spoke of men having to be strong in the wrist to use hand pressure. I use hand pressure and I believe I get my gold so dense that a plugger would not make much of a dent in it. I use smooth points. I use no serrated pluggers with either hand pressure or with the mallet. My pluggers are all smooth; the gold possesses that peculiar quality of cohesion which does not necessitate the use of serrations on instrument to make it cohesive.

Porcelain I use a little. Out in the country we have to do everything. I think of porcelain, like all others who use it, that it has some great advantages over anything else, particularly in incisors where a tooth is decayed very close to the cutting edge. There, with porcelain, it is not necessary to remove the incisal edge, where with gold you could not possibly put in the filling without knocking off the corner; if ever you have filled them with this kind of cavity you have filled them with cement, and they last for years with refilling. The corner remains; by using porcelain in those cases you do not have to cut through to the incisal surface.

I am confident most of us use amalgam empirically. I do not think there is one out of fifty of us that knows what we are doing when we put in an amalgam filling. It seems to me that the sooner I get the amalgam filling into the cavity after having rubbed up the alloy, so as to have the initial set of it take place in the cavity that I get the best results possible.

So far as polishing fillings is concerned, there is no question about that, but there must be complete restoration of contour in all cases. In gold fillings in disto-occlusal cavities of molars and bicuspids, I think it is almost impracticable to put the gold to the mesial or pulpal wall of such cavities with the mallet. I believe it must be done by hand pressure, unless one uses one of those back-action automatics, although I do not know that I ever struck a piece of gold with one in my life. I do not know anything about the use of automatic mallets, but the hand pressure, with smooth plugger points and cohesive gold come pretty near to being the only way to put in gold fillings. The mallet can be used with smooth points.

Dr. M. T. Watson: The automatic has been roasted pretty hard this afternoon. The trouble is that we do not know how to fill teeth with the automatic. I think you can fill any tooth with the automatic that you can with anything else and you do not need to chip the edges either; if you use proper care and prepare the edges right and understand the manipulation with the automatic plugger you can use it all right in any tooth, however frail.

Dr. J. L. Sweetnam: I was asked to read a paper on gold as a filling material. I did not say anything particularly about amalgam or porcelain; my subject was gold. I am a user of amalgam and have been since my early practice. Last year in the mouth of Dr. Henry Brophy, of Chicago, now a dentist, I saw amalgam fillings that I had put in for him 25 years ago. Out of twelve fillings there were ten of them still doing service. I do not try to put amalgam in every place. Like porcelain it requires a good deal of skill to produce nice results. The porcelain inlay is a failure I believe. I cannot afford the time to do this work all over the mouth. I feel that we are running the chance of having to re-cement those fillings in time, or of making new ones.

In regard to the age that gold should be used, I know a great many are putting in gold fillings for children, and I have watched it carefully. Quite a number of years ago I was led to abandon gold in childrens' teeth and I put the limit at 18 years; now I would make it twenty years.

When patients apply to have their mouths put in order, the very first thing is to get their mouth in a healthy condition. We can not work on teeth comfortably to the patient, or to ourselves, with the mouth in a filthy condition. The porcelain inlay probably has a place all right. I have put forth as much effort as any man, of my age at least, in the effort to use porcelain. I have certainly used a great deal of amalgam; but I am very careful in making amalgam fillings. With large fillings in the back of the mouth, and also nearly all amalgam fillings, I have found it to advantage to use cement with it, mixing it quite soft; when the cavity is partially filled I then take out the cement and make sure that there is no cement around the margin of the filling, and then go ahead and finish the filling with amalgam. The amalgam does not discolor the margin of the tooth. I still contend that all materials that we use in teeth, gold has the widest range of usefulness.

Dr. H. L. Raymond: In regard to what Dr. Jackson said, for instance that a filling is not any stronger than its weakest point, of course the cement is the weakest point of a porcelain filling, and that fact in a way makes it the strongest point, for, Dr. Byram says, you cannot have decomposition take place, for if the cement gives way your inlay gives way and it comes out. Porcelain fillings do come out, and very frequently gold fillings come out, and when they do come out you have to enlarge the cavity, whereas porcelain filling can be replaced in a very few minutes. If there is one thing more than another that would make more in favor of porcelain, it is its value in prophylaxis. I believe with you that it is impossible to get a perfect surface with a metal filling, especially a gold filling. You can get a perfect surface with a porcelain filling; it does not tarnish nor take in any accumulations of any kind, but keeps perfectly clean. I notice many good operators are coming to the porcelain filling. Dr. Hoff, quite a few years ago, was not of the same mind that he is to-day. I believe porcelain can be used in a back tooth where anything else can be used, and generally to the best advantage. Dr. Collins spoke about men going to the extremes. We need some men in the extremes to keep us from standing still.

